# EHMTI-0365. The effectiveness of health training on improving sleep quality, and reduction of the symptoms of migraine headaches in individuals with multiple sclerosis

**DOI:** 10.1186/1129-2377-15-S1-J14

**Published:** 2014-09-18

**Authors:** N Torabzadeh, S Asadnia, F Sepehrian Azar, M Zamanlu, NAVA Mohammadi

**Affiliations:** 1 Psychology Department, Urmia University of Medical Sciences, Urmia, Iran; 2 Psychology Department, Urmia University, Urmia, Iran; 3 Psychology Department, Neuroscience Research CenterTabriz University of Medical Sciences, Tabriz, Iran; 4 Psychology Department, Urmia University, Urmia, Iran

## Introduction

Migraine headaches and undesirable quality of sleep in patients with multiple sclerosis are very common.

## Aims

The aim of the present study is the effectiveness of sleep health training on improving sleep quality, and reduction of the symptoms of migraine headaches in individuals with multiple sclerosis.

## Methods

Therefore, to do this, 60 patients with MS peered selected and randomly put into two groups of experimental and control. They answered Pittsburg quality of sleep of the Najarian symptoms of migraine headache. Experimental group took part for four sessions of Sleep health training session. After completing the sessions both answered to two tests again.

## Results

The results showed that sleep health training on improving the quality of sleep and reduction the symptoms of migraine headache has been effective in experimental group.

## Conclusions

Therefore, sleep health training to improve the quality of sleep and reduce the symptoms of migraine headache sufferers of MS, can be conducted along with other pharmaceutical and medical treatments.

No conflict of interest.

